# Medicare Advantage benefits design and access to surgeons

**DOI:** 10.1093/haschl/qxaf112

**Published:** 2025-05-30

**Authors:** Jessica I Billig, Michael Wu, Elsa Zhang, Changchuan Jiang, Joshua M Liao

**Affiliations:** Department of Plastic Surgery, University of Texas Southwestern Medical Center, Dallas, TX 75390, United States; Department of Internal Medicine, Division of General Internal Medicine, University of Texas Southwestern Medical Center, Dallas, TX 75390, United States; Department of Internal Medicine, Division of General Internal Medicine, University of Texas Southwestern Medical Center, Dallas, TX 75390, United States; Department of Internal Medicine, Division of Hematology and Oncology, University of Texas Southwestern Medical Center, Dallas, TX 75390, United States; Peter O’Donnell Jr. School of Public Health, University of Texas Southwestern Medical Center, Dallas, TX 75390, United States; Department of Internal Medicine, Division of General Internal Medicine, University of Texas Southwestern Medical Center, Dallas, TX 75390, United States; Peter O’Donnell Jr. School of Public Health, University of Texas Southwestern Medical Center, Dallas, TX 75390, United States

**Keywords:** Medicare Advantage, benefit design, cost-sharing, surgical access, ancillary benefits, health policy

## Introduction

Out-of-pocket costs (ie, cost-sharing) can deter patients from seeking surgical care, which results in worse quality of care and outcomes compared with patients with greater access.^1,2^ Specifically, high out-of-pocket expenses for surgical care is associated with greater delays in seeking a surgeon, preventable hospitalizations after surgery, and mortality rates.^2-4^ Moreover, patients are bearing more surgical costs with rising out-of-pocket expenses, highlighting an urgent need for effective approaches to combat financial barriers to surgery.^5^

Therefore, strategies that reduce patient cost-sharing could decrease financial barriers for surgical care, thus improving care quality and patient outcomes. Given that out-of-pocket expenses for surgical care are increasing over time, Medicare Advantage (MA), the predominant form of insurance for older Americans, offers reduced cost-sharing for surgery. However, little is known about the extent to which MA plans across the United States offer reduced cost-sharing for surgery. This study evaluates trends over time in for MA plans with reduced cost-sharing for surgery and their county-level distribution in the United States.

## Methods

We conducted a cross-sectional study of US counties using 2022–2024 Centers for Medicare and Medicaid Services Plan Benefits Package and MA enrollment files data, which included county-level information about the number of, enrollment in, and type of (Health Maintenance Organization [HMO]; Preferred Provider Organization [PPO]) MA plans offering reduced cost-sharing for surgery. We defined plans with reduced cost-sharing for surgery if they either offered a reduction in coinsurance or copayment for general surgeons (Appendix Methods).

We assessed MA plans offering reduced cost-sharing for surgery over time (2022–2024) and the changes in type of MA plans. We then categorized and mapped county-level enrollment in these MA plans with reduced cost-sharing for surgery into quartiles (quartile 1 = lowest, quartile 4 = highest) (Appendix Methods). This study was determined to be non-human subjects research by the University of Texas Southwestern's institutional review board and thus exempt.

## Results

Among 6423 MA plans in our sample, the proportion offering reduced cost-sharing for surgery increased from 1.3% in 2022 to 1.8% in 2024. In turn, the number of individuals enrolled in such MA plans increased from 1.1 million individuals in 2022 to 1.3 million individuals in 2024. By comparison, the number of individuals enrolled in other MA plans that did not offer reduced surgical cost-sharing increased from 28.8 million to 33.9 million individuals over the same period (Table 1).

**Table 1.** Quarterly breakdown of Medicare Advantage plans and reduced cost-sharing plans for surgery.

	2022		2023			2024		
	Q2	Q3	Q2	Q3	Q4	Q1	Q3	Q4
All plans								
No. of plans	6453	6443	6908	6908	6908	6809	6807	6808
HMO, % (No.)	64 (4151)	64 (4141)	62 (4305)	62 (4305)	62 (4305)	61 (4139)	61 (4138)	61 (4138)
PPO, % (No.)	36 (2302)	36(2302)	38 (2603)	38 (2603)	38 (2603)	39 (2670)	39 (2669)	39 (2670)
Total enrollment	28 831 115	29 175 571	31 330 492	31 664 242	31 906 257	33 195 293	28 792 726	33 948 259
Surgery								
No. of plans	86	86	95	95	95	125	125	125
HMO, % (No.)	60 (52)	60 (52)	56 (53)	56 (53)	56 (53)	43 (54)	43 (54)	43 (54)
PPO, % (No.)	40 (34)	40 (34)	44 (42)	44 (42)	44 (42)	57 (71)	57 (71)	57 (71)
Total enrollment	1 144 355	1 147 778	1 238 588	1 248 919	1 250 713	1 322 020	1 198 211	1 321 530
No. with reduced coinsurance	86	86	94	94	94	124	124	124
No. with reduced copayment	0	0	1	1	1	1	1	1

Abbreviations: HMO, Health Maintenance Organization; PPO, Preferred Provider Organization; Q, quartile.

Among MA plans offering reduced cost-sharing for surgery, the proportion of PPOs increased (from 40% of plans in 2022 to 57% in 2024), while the proportion of HMOs decreased (from 60% of plans in 2022 to 43% in 2024). Among other MA plans, the proportion of PPOs slightly increased (from 36% to 39%), while the proportion of HMOs slightly decreased (from 64% to 61%). The MA plans with reduced cost-sharing for surgery were offered almost entirely through co-insurance, with only 1 plan offering copayment reduction during our study period (Table 1).

**Figure 1.**
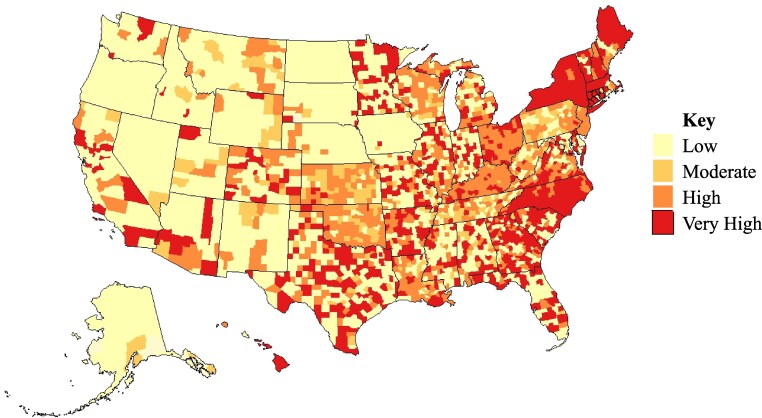
Medicare Advantage (MA) enrollment for plans offering reduced cost-sharing for surgery. We created quartiles for MA enrollment for plans offering reduced cost-sharing for surgery per 1000 MA enrollees using 2022 MA enrollment files to understand financial access to surgeons on the county level.

There was heterogeneity in county-level MA enrollment in reduced cost-sharing for surgery (Figure 1). Of 3225 US counties, most had low MA enrollment in plans offering reduced surgical cost-sharing (1355 counties; 42.02%).

## Discussion

In this national analysis of US counties, there were modest increases in the number of and enrollment in MA plans offering reduced cost-sharing for surgery. We also found heterogeneity in enrollment in MA plans with reduced cost-sharing for surgery.

The increase in the number of MA plans with reduced cost-sharing for surgery may reflect that insurance plans are attempting to decrease financial barriers for surgery. Surgical care is expensive, and these financial barriers can lead to differential outcomes after major surgery.^1,3,6^ Therefore, benefits such as reducing cost-sharing for surgery may be 1 avenue to reduce financial barriers for surgical care. However, we found a very modest increase in the enrollment in these reduced cost-sharing plans, highlighting that cost-sharing may not be the predominant driver for patient selection of MA plans.

Beyond these financial barriers, physical barriers and other insurance-related barriers also have a profound impact on surgical outcomes. Physical barriers, such as travel distance for surgical care, can lead to patients experiencing differential surgical treatment, delays in their care, and worse outcomes compared with patients who have limited travel time.^6-8^ Additionally, other insurance-related barriers besides out-of-pocket expenses such as utilization management strategies, particularly prior authorization, can also result in differential surgical treatment and surgical delays.^9,10^ Therefore, financial incentives such as reduction in cost-sharing for surgery are only 1 piece of the solution for improving access to surgical care.

Study limitations include a cross-sectional design and lack of data on provider networks or patient utilization. Nonetheless, our results show early trends in enrollment in insurance plans that reduce patients' out-of-pocket costs and geographic heterogeneity in these insurance benefits. Additional strategies may be necessary to remove financial deterrents to surgical care and improve access to surgical care.

## Contribution statement

J.I.B. and J.M.L.: study concept and design. J.I.B., M.W., E.Z., C.J., and J.M.L.: acquisition, analysis, or interpretation of data; critical revision of the manuscript for important intellectual content; statistical analysis; and study supervision. J.I.B. and E.Z.: drafting of the manuscript. J.I.B. and J.M.L. had full access to all the data in the study and take responsibility for the integrity of the data and the accuracy of the data analysis.

## Supplementary Material

qxaf112_Supplementary_Data

## Data Availability

Data is publicly available on the Centers for Medicare and Medicaid Services website.
