# Supplementary material for: Medicare Advantage benefits design and access to surgeons
Source: Health Aff Sch. 2025 May 30;3(6):qxaf112. doi: 10.1093/haschl/qxaf112 (PMC12152724; doi:10.1093/haschl/qxaf112)
Supplement: qxaf112_Supplementary_Data [file qxaf112_supplementary_data.zip › 051925_MA_Surgeon_Appendix.docx]

**Appendix Methods**

*Centers for Medicare and Medicaid Services (CMS) Data Source*

We collected all available CMS Benefits Medicare Advantage/Part Contract and Enrollment data from 2022 second quarter through 2024 fourth quarter by contract, plan and county level. These data are publicly available Medicare Advantage plan benefit design from CMS.

*Cost-Sharing Definitions*

Cost-sharing data was collected under the relevant CMS files where plans indicated inclusion of value-based insurance design for general surgery under medical specialists. Reduced cost-sharing offered by plans was defined as being inclusive of the following categories: general surgeons with reduced copayment, coinsurance, or deductible along with their respective covered percent and dollar amounts. However, there were zero MA plans with reduced deductible for general surgeons.

*MA Quartile Splits*

For plans offering reduced cost-sharing for general surgery, the Federal Information Processing Standards (FIPS) county code was utilized to identify plan coverage area and the corresponding number of MA enrollees for each plan covering each county. We then divided the number of MA enrollees for all US counties by per 1000 enrollees and assigned them into the following: 1^st^ quartile as lowest to under 25% of all enrollee counts, 2^nd^ quartile as 25% to under 50% of all enrollee counts, 3^rd^ quartile as 50% to under 75% of all enrollee counts, and 4^th^ quartile as 75% to highest enrollee counts.
